# Reduced contrast sensitivity function is correlated with changes to cone photoreceptors in simple high myopia

**DOI:** 10.3389/fnins.2024.1274651

**Published:** 2024-03-22

**Authors:** Jiefang Wang, Xinting Liu, Jing Huang, Ruoyun Deng, Sijun Zhao, Yulei Chen, Zhaohe Chen, Yanli Wang, Yu Rong, Qian Liu, Jia Qu, Xinjie Mao

**Affiliations:** ^1^Eye Hospital and School of Optometry and Ophthalmology, Wenzhou Medical University, Wenzhou, Zhejiang, China; ^2^State Key Laboratory of Ophthalmology, Optometry and Vision Science, Eye Hospital, Wenzhou Medical University, Wenzhou, China; ^3^National Clinical Research Center for Ocular Diseases, Eye Hospital, Wenzhou Medical University, Wenzhou, China

**Keywords:** adaptive optics, contrast sensitivity function, optical coherence tomography, photoreceptor degeneration, simple high myopia

## Abstract

**Purpose:**

To investigate the contrast sensitivity function (CSF) changes in simple high myopia (SHM) and evaluate the correlations between these changes with the early changes in the retinal microstructure.

**Methods:**

This prospective study comprised 81 subjects, 20 with emmetropia (EM), 26 with low myopia and moderate myopia (LM/MM), and 35 with SHM. The area under the log CSF curve (AULCSF) and the cut-off spatial frequency (Cut-off SF) were employed as measures of CSF. Adaptive optics (AO) was employed to quantify the cone density, spacing, and regularity. The thickness and blood flow of the retinal sublayers were determined from vertical and horizontal optical coherence tomography angiography (OCTA) A-scans. Swept-source optical coherence tomography (SS-OCT) was employed to analyze the choroidal thickness (CT) and choroidal vascularity using a custom algorithm. Differences in the retinal and choroidal parameters, cone distribution, AULCSF, and Cut-off SF were compared among the three groups. Multivariate linear mixed models were used to elucidate the associations between photoreceptor morphological alterations, retinal and choroidal parameters, and AULCSF.

**Results:**

The AULCSF and Cut-off SF were significantly lower in the SHM group compared to the EM and LM groups (*p* < 0.05). The SHM group had less cone density, larger cone spacing, and lower cone regularity than the EM and LM/MM groups (*p* < 0.05). Moreover, the thickness of the inner segment of photoreceptors (IS), retinal pigment epithelium (RPE) layer and choroid were reduced, and the outer segment of photoreceptors (OS) was thicker in the SHM group compared to the EM and LM/MM groups (all *p* < 0.05). A longer axial length (AL) was correlated with decreased AULCSF, cone density, and cone spacing (*r* = −0.800 to 0.752, all *p* < 0.050). Additionally, decreased CSF was correlated with lower cone density (*r* = 0.338, *p* = 0.035).

**Conclusion:**

Decreased contrast sensitivity was observed in patients with SHM and cone density was significantly correlated with reduced AUCSF.

## 1 Introduction

Myopia is increasingly becoming common globally. By the year 2050, it is estimated that 10% of the global population will have high myopia (HM), which translates to nearly one billion people (Holden et al., 2016). The pathological changes that occur due to HM can cause serious visual impairment, even blindness (Haarman et al., 2020). According to the literature, up to 70% of patients with HM have some degree of ocular pathology, which can seriously affect the patient’s vision and life (Wong et al., 2000; Ohno-Matsui and Jonas, 2019). Therefore, measures to prevent the progression of myopia, ocular complications, and vision loss due to HM are urgently needed.

Simple high myopia (SHM) is characterized by a refractive error of ≤ −6.00 D, with no systemic or ocular complications, and the degree tends to stabilize in adulthood. Clinically, SHM patients exhibit no pathological change or structural damage to the macular region, which makes it simple to overlook the structural and functional changes (Xu et al., 2007; Verkicharla et al., 2015). Contrast sensitivity, which is defined as the inverse of the contrast of the sensory threshold of the human eye at different spatial frequencies, provides a better evaluation of the visual function than the standard visual acuity chart examination commonly used in clinical practice, and can also better reflect practical visual acuity in daily life (Liou and Chiu, 2001; Richman et al., 2013). It has been reported in the literature (Liu et al., 2022) that contrast sensitivity was reduced in patients with HM. In addition, the contrast sensitivity function (CSF) can provide a comprehensive assessment of the spatial vision across different contrast conditions and spatial frequencies (Chatzistefanou et al., 2005; Joltikov et al., 2017; Alahmadi et al., 2018). For this reason, in this work, contrast sensitivity was measured in patients with SHM to capture their state of visual function.

The advancements in high-resolution imaging techniques, such as adaptive optics (AO) and optical coherence tomography (OCT) have made non-invasive imaging and *in vivo* analysis of the retinal structure possible (Li et al., 2010; Flores-Moreno et al., 2013; Park et al., 2013; Liu et al., 2015). AO can distinguish cones and rods in the retina and provides a view of the retinal vasculature at a high resolution (Akyol et al., 2021). Wang et al. (2019) demonstrated a strong agreement between AO and OCT imaging in detecting retinal microstructure. This finding clearly indicated that AO and OCT can be used to detect the cone photoreceptors objectively and accurately, and more importantly, it can be performed in a relatively short time.

Many studies have shown that SHM causes microstructural changes within the eye. For example, almost synchronous myopia-related retinal microvascular reductions in both the superficial and deep retinal layers have been identified (Lam et al., 2007; Liu et al., 2021). In addition, Qiuyan Wu et al. reported thinning of the retinal (RT)/choroidal thickness (CT) and decreased retinal vessel density (Wu et al., 2020). Some works in the literature have also reported that the distribution of retinal cone photoreceptor cells is an important factor in visual impairment (Cheng et al., 2021). A thorough investigation of the changes in the microscopic structure of the retinal choroid can provide valuable insights into the decrease in visual function of people with SHM.

However, many current works only focus on the changes in the retinal microstructure and visual function in people with SHM, while the correlation between the retinal microstructure and function has been scarcely reported in the literature. Therefore, further studies are undoubtedly needed to determine the specific causes of visual dysfunction due to changes in the retinal microstructure. Thus, the purpose of this work was to determine whether the changes in the microstructure of the retina and choroid in individuals with SHM are associated with CSF.

## 2 Methods

### 2.1 Subjects

All subjects in this cross-sectional study were recruited from the Eye Hospital of Wenzhou Medical University between May 2020 and October 2020. The study protocol followed the principles of the Helsinki Declaration and was approved by the Research Review Board of Wenzhou Medical University’s Eye Hospital. Before starting the experiment, each subject provided written informed consent. The protocol for the study was submitted to the Chinese Clinical Trials Registry (Registration No. ChiCTR2000040926).

Before formal enrolment in the study, ophthalmic screening examinations, such as non-cycloplegic subjective refraction, binocular testing, ocular health evaluation, AL, and intraocular pressure (IOP) measurement, were performed. Subjects who had astigmatism more than −1.00 diopter (D), IOP more than 21 mm Hg, BCVA worse than 20/20, significant cataracts, diabetic retinopathy, age-related macular degeneration, glaucoma, a history of intraocular surgery, serious complications of HM such as retinoschisis and choroidal neovascularization, and those with related systemic diseases were excluded from the study. Caffeine and alcohol consumption was prohibited for 24 h before choroidal imaging.

The enrolled subjects were divided into the following three groups: (1) a control group with a spherical equivalent (SE) ranging from −1.00 to +1.00 D, (2) a low to moderate myopia group with myopia between −1.50 and − 6.00 D, and (3) SHM group with a SE exceeding or equal to −6.00 D or an axial length (AL) ≥ 26.5 mm, and without myopic maculopathy.

### 2.2 Adaptive optics measurements

**Figure 1:**
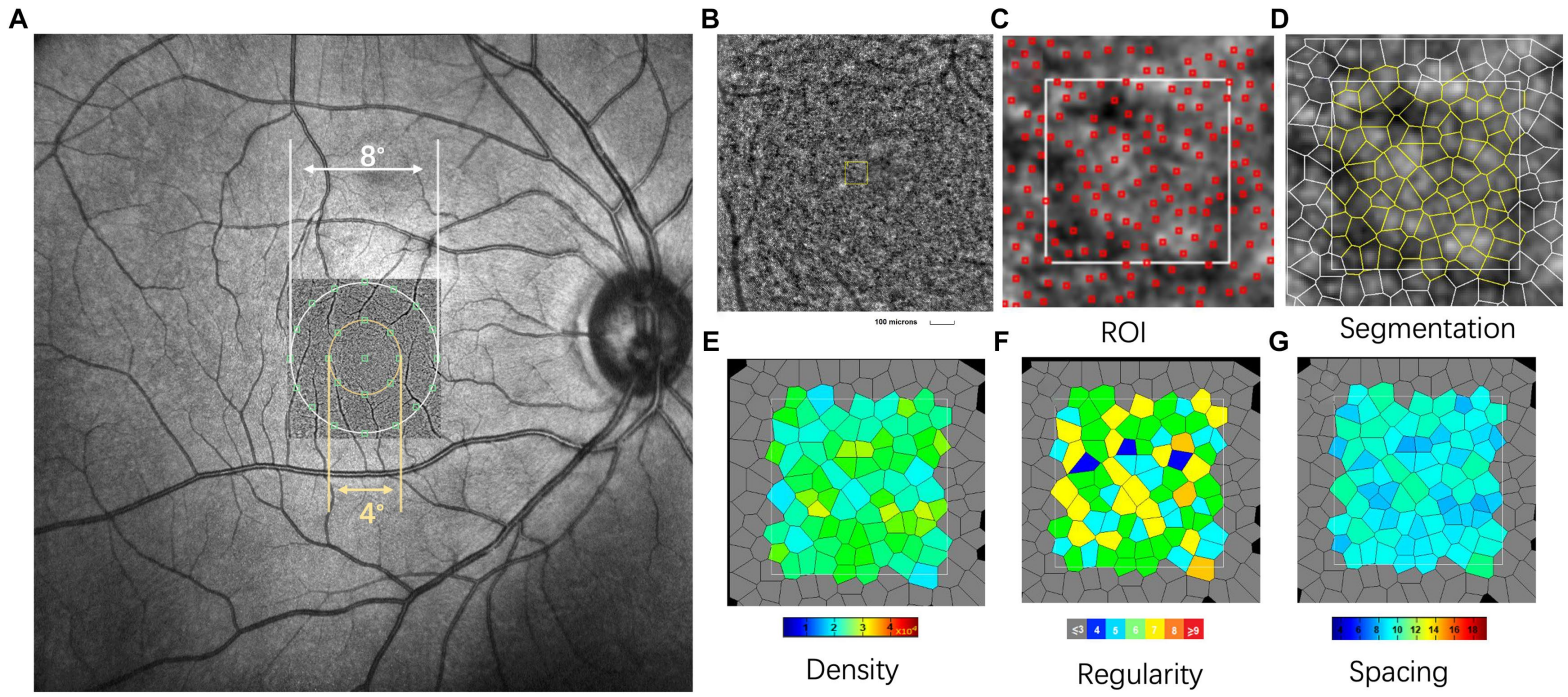
Representative images of swept-optical coherence tomography angiography (SS-OCTA) and adaptive optics (AO). **(A)** The AO image is superimposed on the fundus image by SS-OCTA. The AO montage with a window size of 10^°^ × 10^°^ was created centered on the fovea. The eight green squares shown in the figure represent the measured locations. **(B)** The white square is a magnified view of a region of interest (ROI) (white square, represented acquisition window (the size of 100 × 100 μm)). **(C,D)** Detection and boundaries segmentation of cone photoreceptor using automatic software (AO detect 2.0b13, Imaging Eyes). Red squares indicate the positions of cones used to assess cone distribution. **(E–G)** Represented the cone density regularity and cone spacing of closest neighbors. In this single case with AL of 23.55 mm, 78 cone photoreceptors were detected in ROI. The cone density, regularity and spacing were 20,709/mm^2^, 94.6%, and 5.45 μm, respectively. N, number of cone cells.

An AO fundus camera (rtx1, Imagine Eyes, Orsay, France) was used to observe the spatial features of cone photoreceptors. The apparatus included a Shack-Hartmann wavefront sensor, a deformable mirror, and a high-resolution fundus camera. The lateral resolution was 1.6 μm with an approximate 4.2^°^ × 4.2^°^ field of view. During imaging, the subjects were asked to gaze at an inbuilt yellow cross target that could be moved by the experimenter to pre-set coordinates within ±10^°^ horizontally and ± 8^°^ vertically. During image acquisition, the images that clearly showed a cone mosaic pattern were recorded. After the acquisition of a video, including a series of 40 frames at each location, a built-in program correlated and averaged the captured frames to produce a final image with a window size of approximately 4.0^°^ × 4.0^°^. In the current study, the initial coordinate was first captured, corresponding to the fovea. The subjects were asked to consecutively fixate at 3^°^ of eccentricity along the four meridians (superior, temporal, inferior, and nasal). Following the acquisition of the images, nine pictures were analyzed with the system’s built-in software (i2k Retina Pro) to generate a montage of approximately 10^°^ × 10^°^, which was equivalent to 3 × 3 mm (Figure 1A).

**Figure 2:**
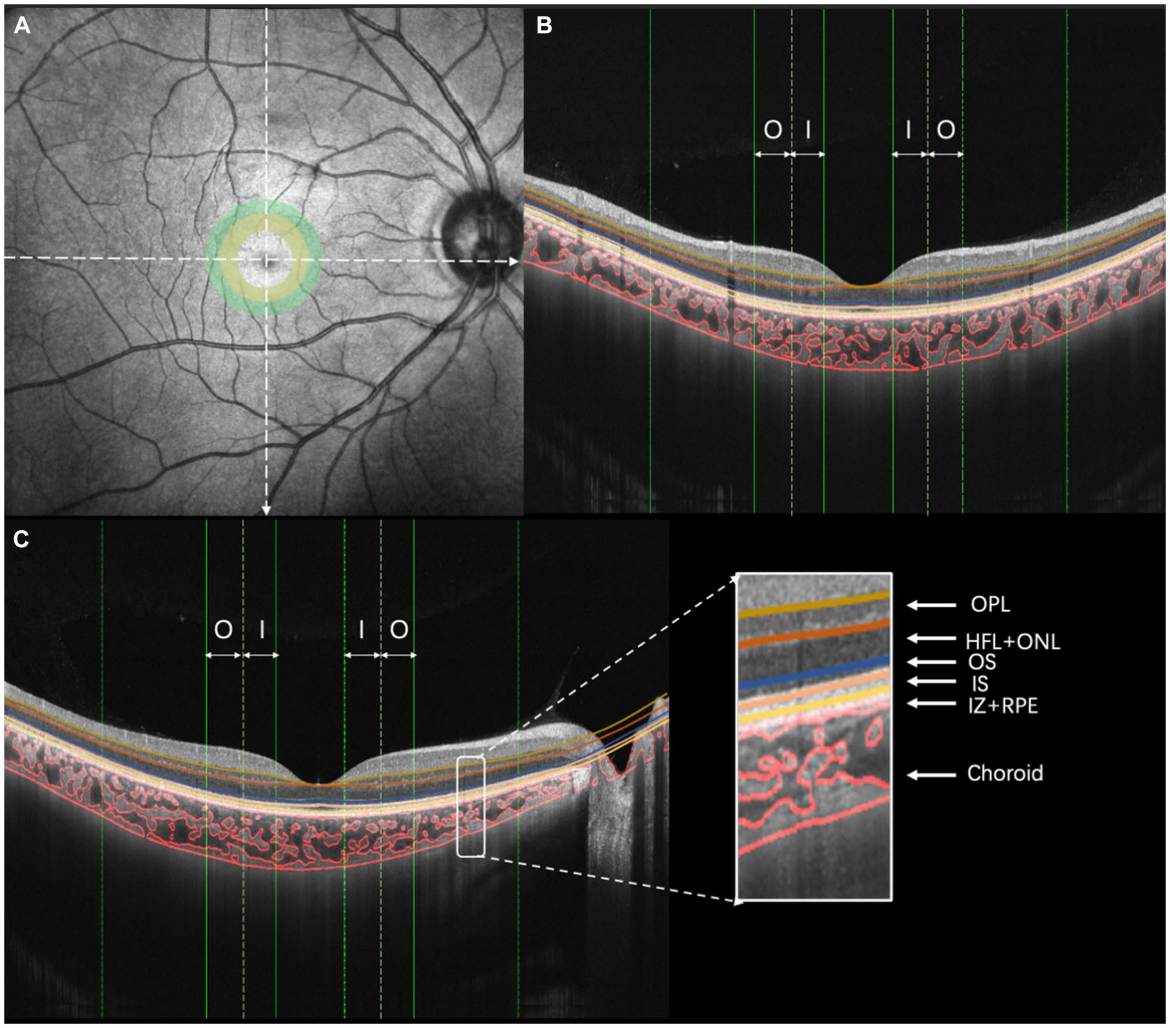
Illustration of choroidal vasculature analysis and retinal layering. **(A)** OCT image, **(B)** vertical scan, and **(C)** horizontal scan [I, inner region (ranging from 0.5 to 1.0 mm of eccentricity); O, outer region (ranging from 1.0 to 1.5 mm of eccentricity)].

The spatial features of the cone photoreceptors were analyzed using software provided by the manufacturer (AO Detect 0.2; Imaging Eyes, Orsay, France). The cones were analyzed at 2^°^ and 4^°^ eccentricities along the four meridians (superior, temporal, inferior, and nasal). After correcting the magnification of the montage to ensure a magnification that was similar to that used for the OCT images, three 100 × 100 μm regions of interest (ROIs, Figure 1B) were selected to avoid blood vessels. These ROIs were selected from locations at 0.6 mm and 1.2 mm eccentricity along each meridian (corresponding to the inner and outer regions, respectively; Figure 2A). A built-in algorithm corrected the magnification of the size of the ROIs based on the AL. Then, segmentation and Delaunay triangulation algorithms automatically identified the cone photoreceptors (Figures 1C,D), and the corresponding density, spacing, and regularity were then calculated (Figures 1E–G). Cone density was defined as the number of cells per square millimeter; cone spacing was defined as the center-to-center spacing of adjacent cones; cone regularity was defined as the percentage of cells that had five to seven neighbor cells. The average values of the three parameters were then obtained.

### 2.3 OCT image acquisition and analysis

A swept-source optical coherence tomography (SS-OCT)/OCT-angiography (OCTA) system (VG200S; SVision Imaging, Henan, China) equipped with an SS laser that had a central wavelength of approximately 1,050 nm and a scan rate of 200,000 A-scans per second was used for image acquisition. Structural OCT of the macular region was performed with 18 radial scan lines centered on the fovea. Each scan line, generated by 2048 A-scans, was 12 mm long and separated from the adjacent lines by 10°. Only the vertical and horizontal scans were used to analyze the CT and choroidal vascularity (Figure 2). The choroid in the SS-OCT images was defined as the area from the retinal pigment epithelium (RPE)–Bruch’s membrane complex to the choroid–sclera interface. After semiautomatic choroidal segmentation with a custom algorithm developed in MATLAB R2017a (MathWorks, Natick MA, USA), segmentations of RPE–Bruch’s membrane complex and choroid–sclera interface were adjusted manually by a trained examiner (JW). Bennett’s formula adjusted the scan size to accommodate variations in magnification caused by different ALs among the eyes (Littmann, 1982; Bennett et al., 1994; Kang et al., 2010). Bennett’s formula relies on the AL to correct ocular magnification, which is approximated based on the estimated location of the second principal point and its usual spatial relationship to the nodal point (Liu et al., 2015). After segmentation, each image was linearized using custom-designed algorithms in MATLAB R2017a to demarcate the luminal area (LA) and stromal area (SA) according to Niblack’s auto-local threshold; this method was first proposed by Sonoda et al. (2015) and was further developed by Agrawal et al. (2016). After performing image processing, the mean macular CT and choroidal vascular LA were calculated. The choroidal vascularity index (CVI) was defined as the ratio of the LA to the total choroidal area (TCA).

The retinal thickness and blood flow measurements for all subjects were performed based on OCTA (Optovue RTVue XR Avanti; Optovue, Inc., Fremont, CA, USA). The RTVue OCT scanning speed was 70,000 A-scans per second and the wavelength of the light source was 840 nm with a 50 nm bandwidth. Each OCTA image was composed of 304 pixels in the horizontal and vertical directions. A signal strength index greater than 50 indicates that the center of the scan is well aligned and the image can be used for further analysis (Garas et al., 2010). The OCTA scan area was centered on the fovea with a 3 mm × 3 mm field of view. The first subfield was the inner region, ranging from 0.5 to 1.0 mm eccentricity (Figure 2A, yellow circle). The second subfield was the outer region, ranging from 1.0 to 1.5 mm eccentricity (Figure 2A, green circle). The thicknesses of the following fundus layers were quantified: outer plexiform layer (OPL), Henle fiber layer and outer nuclear layer (HFL + ONL), outer segment of photoreceptors (OS), an inner segment of photoreceptors (IS), interdigitation zone and retinal pigment epithelium/Bruch complex (IZ + RPE). All of the detected intraretinal layer boundaries were segmented using graph theory and the shortest-path search method based on an optimization algorithm of the dynamic programming technique (Chiu et al., 2010; Liu et al., 2014).

To ensure consistent agreement among examiners, two trained examiners (JW and JH) segmented twice all the boundaries. Intraclass correlation coefficients (ICCs) and coefficients of repeatability were calculated to assess the agreement of ChT, LA, SA, TCA, CVI, OPL, HFL + ONL, OS, IS, and IZ + RPE measurements within and between examiners at various regions. The coefficient of repeatability was calculated as 1.96 times the standard deviation of the differences between two measurements. After obtaining good agreement, all the scans were measured by examiner JW. To reduce the effect of diurnal variation on the choroid and ensure the consistency and accuracy of the results, all measurements were taken between 13:30 and 17:00 (Tan et al., 2012).

### 2.4 Contrast sensitivity function (CSF)

All tests were carried out in a dark room using a visual function test workstation (Zhishiyuan, JH-P02, Model NO.102JST190828001, Jiangsu Juehua Medical Technology Co., Ltd.). The workstation comprised a personal computer (PC) and a monitor. The PC generated and controlled the stimuli, which were then displayed on the monitor. The monitor had a resolution of 2,560 × 1,440 pixels, a refresh rate of 60 Hz, and an average luminance of 74.5 cd/m^2^. The stimulus was a sinusoidal grating with a spatial frequency of 1.5, 3, 6, 12, 18, and 24 cycles per degree (cpd) at a viewing distance of 2 m. To reduce the edge effect, a 0.5^°^ Gaussian ramp was added around the stimulus. The subjects were first introduced to the entire experiment, including the visual task, prior to the test. The CSF was performed with spectacle correction under best corrected Rx and took about 25 min. A brief beep prompted the start of the trial, together with the presentation of a crosshair (3.0 × 3.0^°^) to indicate the location of the stimulus. After 150 ms, the cross disappeared, and the stimulus grating of vertical or horizontal orientation (with equal probability) was displayed for 167 ms. A blank background with a mean luminance of 74.5 cd/m2 was then displayed and the subjects were asked to report the perceived orientation using the keyboard (by pressing the corresponding arrow key). The intertrial interval was 800 ms. A Psi method controlled the grating contrast and separately estimated the contrast threshold that corresponded to 80.3% correct for each spatial frequency. The reciprocal of the contrast threshold was used to calculate contrast sensitivity. There were 270 trials in total, with 45 trials in each spatial frequency. To achieve high-precision grey-scale stimulation, the workstation used the bit-stealing method.

The area under the log CSF curve (AULCSF) is a widely used summary metric of the CSF function (Joltikov et al., 2017; Alahmadi et al., 2018). The cut-off spatial frequency (cut-off SF) characterizes the high-frequency resolution of the visual system. Thus, the AULCSF and cut-off SF of the visual system were analyzed in this study.

### 2.5 Statistical analysis

SPSS was used for statistical analyses (version 22.0; SPSS, Inc., Chicago, IL, USA). To compare the differences between the three groups, one-way ANOVA was performed. The x^2^ test was used to examine the gender distributions of the three groups. Pearson’s correlations and regression analyses were used to determine the relationships between photoreceptor morphologic parameters and both AL and CT. The effects of changes in photoreceptor distribution and density on CSF and other parameters such as the AL and CT were assessed using univariate and multivariate linear regression analyses. *p* < 0.05 was taken to indicate a statistically significant difference between the groups.

## 3 Results

### 3.1 Patient characteristics

A total of 81 eyes were studied, comprising 20 EM eyes, 26 LM/MM eyes, and 35 SHM eyes. The mean SE in the SHM group was −8.00 ± 1.50 D, while the mean SE was −0.15 ± 0.35 D and − 3.65 ± 1.60 D in the EM and LM/MM groups, respectively. Subjects in the SHM group had higher levels of myopia and longer ALs than subjects in the EM and LM/MM groups. The SHM group also had more astigmatism than the EM group (*p* = 0.005, data not shown), but the difference was only 0.46 D. There were no statistically significant differences in age among the three groups (P_age_ = 0.832, Table 1).

**Table 1:** Basic characteristics of the EM/LM, MM, and HM groups.

	EM	LM/MM	HM	*p*
N	20	26	35	-
Sex, F/M	11/9	10/16	11/24	-
Age, y (range)	23.35 ± 1.66 (22 ~ 27)	23.27 ± 1.66 (22 ~ 29)	23.57 ± 2.39 (19 ~ 30)	0.832
SE, D (range)	−0.15 ± 0.35 (−0.75 ~ 0.75)	−3.65 ± 1.60 (−6.00 ~ −0.75)	−8.00 ± 1.50 (−10.50 ~ −4.25)	**<0.001**
AL, mm (range)	23.64 ± 0.69 (22.45 ~ 24.80)	24.76 ± 0.78 (23.31 ~ 27.03)	26.77 ± 1.05 (25.03 ~ 29.23)	**0.036**
IOP, mmHg (range)	14.44 ± 3.16 (9.70 ~ 20)	15.70 ± 2.78 (10.90 ~ 20.50)	16.14 ± 2.57 (12.10 ~ 21)	-

Bold text indicates the specific value of the data for which *p* < 0.05. *p* < 0.05 was taken to indicate a statistically significant difference between the groups.

### 3.2 Differences in CSF among the three groups

The AULCSF of the SHM group was significantly lower than that of the EM and LM/MM groups (AULCSF = 1.55 ± 0.35 in the SHM group, 1.63 ± 0.42 in the EM group, and 1.86 ± 0.24 in the LM/MM group, *p* = 0.003, Table 2). The Cut-off SF in the SHM group was 1.29 ± 0.18 cpd, which was lower than that of the EM group (1.33 ± 0.22 cpd) and the LM/MM group (1.42 ± 0.12 cpd) (*p* = 0.013). The relationship between the AULCSF and AL was significant (*r* = −0.256, *p* = 0.047).

**Table 2:** Contrast sensitivity and cone distribution including cell distribution, cell spacing, and regularity.

	EM	LM/MM	HM	P0	P1	P2	P3
Contrast sensitivity							
AULCSF	1.63 ± 0.42	1.86 ± 0.24	1.55 ± 0.35	**0.003**	0.454	**0.023**	**0.001**
CUTOFF	1.33 ± 0.22	1.42 ± 0.12	1.29 ± 0.18	**0.013**	0.352	**0.087**	**0.004**
Cone distribution							
Cell density,10^3^/mm^2^							
Inner	20.99 ± 1.14	19.16 ± 1.36	16.05 ± 2.62	**<0.001**	**<0.001**	**0.003**	**<0.001**
Outer	21.05 ± 1.45	19.20 ± 1.94	16.56 ± 2.71	**<0.001**	**<0.001**	**0.006**	**<0.001**
Cell spacing							
inner	5.24 ± 0.21	5.27 ± 0.28	5.51 ± 0.40	**0.006**	**0.006**	0.763	**0.009**
Outer	5.25 ± 0.23	5.31 ± 0.34	5.53 ± 0.49	**0.025**	**0.015**	0.598	**0.039**
Regularity	196.43 ± 319.77	90.73 ± 5.05	86.43 ± 5.00	**0.034**	**0.015**	**0.027**	0.916

P0, *p* values between the three groups; P1, *p* values for the emmetropia group compared with the high myopia group; P2, *p* values for the emmetropia group compared with the low to moderate myopia group; P3, *p* values for the high myopia group compared with the low to moderate myopia group. Bold text indicates the specific value of the data for which *p* < 0.05. *p* < 0.05 was taken to indicate a statistically significant difference between the groups.

### 3.3 Differences in the cone distribution among the three groups

The cone distribution was examined using an rtx1-e AO retinal flood-illumination camera (Imagine Eyes, Orsay, France). The density, spacing, and regularity of the spatial cone distribution differed significantly among the three groups (Table 2). The cone density of the HM group was lower than those of the EM and LM/MM groups, in the inner and outer regions (all *p* < 0.001). The cell spacings in the SHM group were significantly increased compared to the EM and LM/MM groups. The cone regularity in the SHM group was significantly lower than those of the other two groups (all *p* < 0.05).

Increases in AL were associated with decreases in the cone density (*r* = −0.731 to −0.705, *p* < 0.001). The associations between the AL and cone spacing were statistically significant (*r* = 0.450 to 0.541, *p* < 0.001, Table 3).

**Table 3:** Linear regression analysis of photoreceptor biomorphology and other variables associated with AL.

Parameters	Univariate linear regression analysis			Multivariate linear regression analysis		
	Unstandardized coefficients beta	Standardized coefficients beta	*p*	Unstandardized coefficients beta	Standardized coefficients beta	*p*
AULCSF	−0.066	−0.256	**0.047**	-	-	-
Cut-Off SF	−0.032	−0.246	0.056	-	-	-
Cone density						
Inner	<0.001	−0.731	**<0.001**	<0.001	−0.516	**<0.001**
Outer	<0.001	−0.705	**<0.001**	-	-	-
Cone spacing						
Inner	1.943	0.541	**<0.001**	-	-	-
Outer	1.353	0.450	**<0.001**	-	-	-
Cone regularity	−0.053	−0.214	0.097	-	-	-
Retinal superficial blood flow						
Inner	0.060	0.362	**0.004**	0.031	0.186	**0.011**
Outer	−0.060	−0.139	0.286	-	-	-
Retinal deep blood flow						
Inner	0.056	0.311	**0.015**	-	-	-
Outer	−0.086	−0.257	**0.045**	-	-	-
FAZ	−4.401	−0.380	**0.003**			
IS						
Inner	−0.357	−0.412	**0.001**	-	-	-
Outer	−0.359	−0.417	**0.001**			
OS						
Inner	0.032	0.101	0.438	-	-	-
Outer	0.075	0.194	0.134			
IZ + RPE						
Inner	−0.073	−0.210	0.104	-	-	-
Outer	−0.113	−0.286	**0.025**	-	-	-
CVI						
Inner	−9.433	−0.305	**0.017**	-	-	-
Outer	−10.018	−0.280	**0.029**	-	-	-
LA						
Inner	−21.798	−0.712	**<0.001**	-	-	-
Outer	−22.357	−0.682	**<0.001**	-	-	-
SA						
Inner	−32.697	−0.676	**<0.001**	-	-	-
Outer	−34.877	−0.683	**<0.001**	-	-	-
TCA						
Inner	−13.955	−0.721	**<0.001**	−7.216	−0.373	**<0.001**
Outer	−14.305	−0.697	**<0.001**	-	-	-
CT						
Inner	−13.845	−0.717	**<0.001**	-	-	-
Outer	−14.333	−0.699	**<0.001**	-	-	-

Bold text indicates the specific value of the data for which *p* < 0.05. *p* < 0.05 was taken to indicate a statistically significant difference between the groups.

### 3.4 Retinal and choroid thickness and blood flow

**Figure 3:**
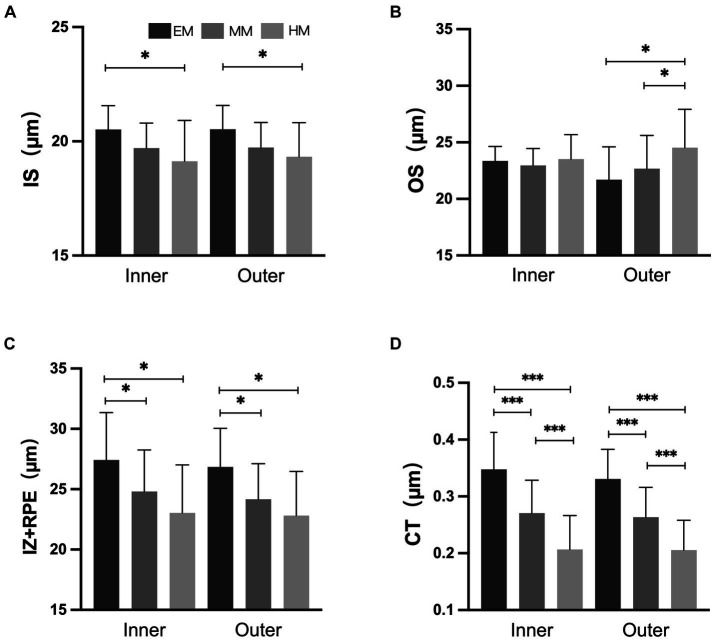
Retinal and choroidal thickness among the three groups. **(A)** Inner segment of photoreceptors (IS); **(B)** outer segment of photoreceptors (OS); **(C)** interdigitation zone and retinal pigment epithelium/Bruch complex (IZ + RPE); **(D)** choroidal thickness (CT). *p < 0.050; **p < 0.010; *** *p* < 0.001.

**Figure 4:**
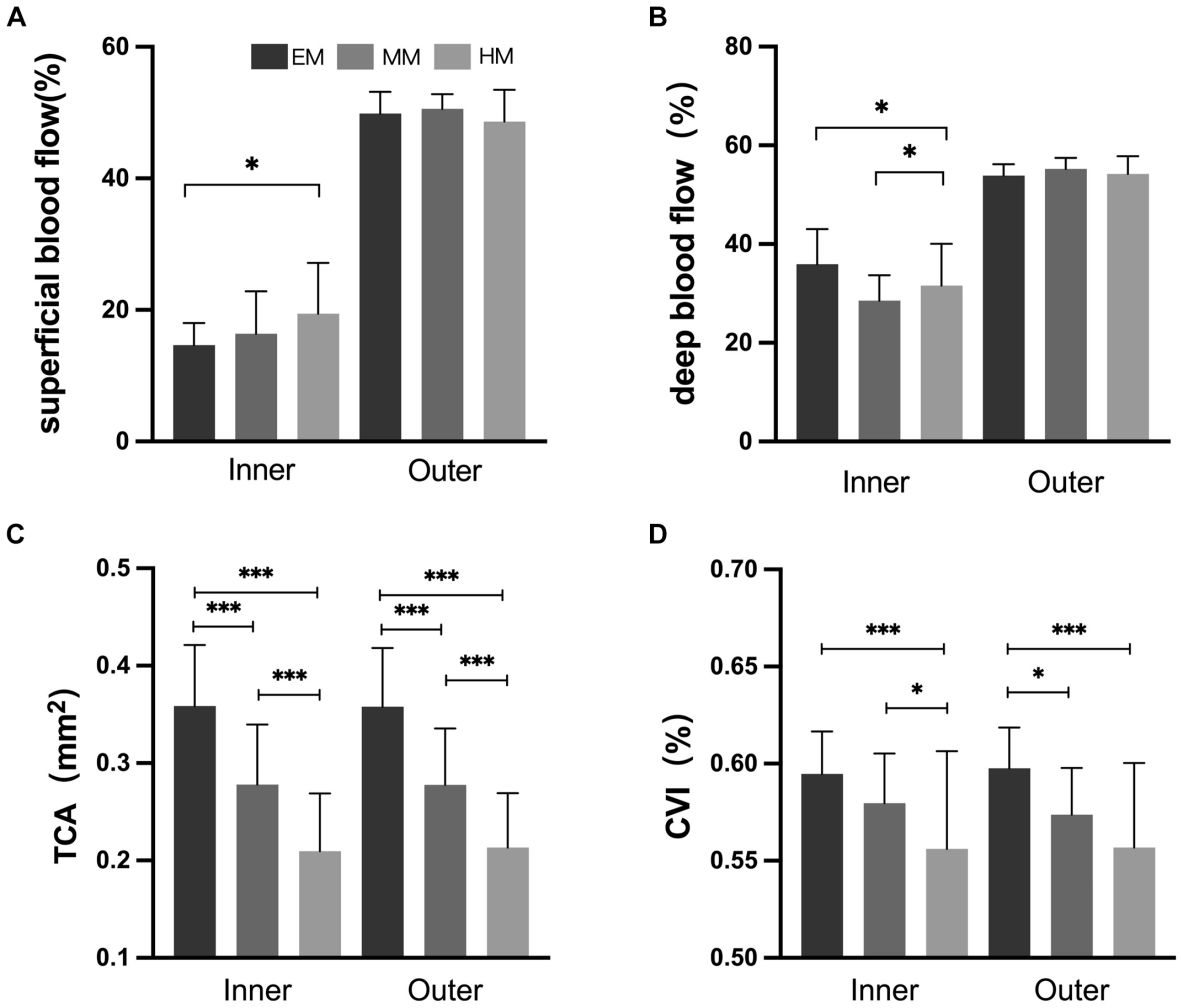
Retinal and choroidal blood flow among the three groups. **(A)** Superficial blood flow; **(B)** deep blood flow; **(C)** total choroidal area (TCA); **(D)** choroidal vascularity index (CVI). *p < 0.050; **p < 0.010; ***p < 0.001.

In the inner and outer regions, the SHM group had a thinner choroid than the EM and LM/MM groups (Figure 3, *p* < 0.001). In the HM group, the TCA and CVI were lower than in the EM group in the inner and outer regions (Figure 4, *p* < 0.001). The SHM group had a thinner LA and SA than the EM and LM/MM groups (*p* < 0.001).

In the inner and outer regions, the IZ + RPE was thinner in the SHM group than in the EM and LM/MM groups. The IS was thinner in the SHM group than in the EM group in the inner and outer regions; however, there were no statistically significant differences between the MM/LM and SHM groups (Figure 3). In contrast, the OS layer of the SHM group was thicker in the outer region compared with the EM and MM/LM groups (*p* < 0.05), whereas there were no significant differences between the EM and MM/LM group. The OPL layer was thinner in the MM/LM group compared to the EM group in the inner region (*p* < 0.05). Differences in the HFL + ONL thickness between the three groups were not statistically significant.

Increases in the AL were associated with reductions in the thicknesses of the IS and IZ + RPE in the outer region, choroid, CVI, LA and SA (*r* = −0.286 to −0.717, all *p* < 0.050). In contrast, the OS layer tended to increase with increasing AL, but this was not statistically significant (*p* > 0.050). The associations between the AL and both the OS and IZ + RPE thicknesses in the inner region were not statistically significant (Table 3).

### 3.5 Multivariate regression model to predict CSF

**Figure 5:**
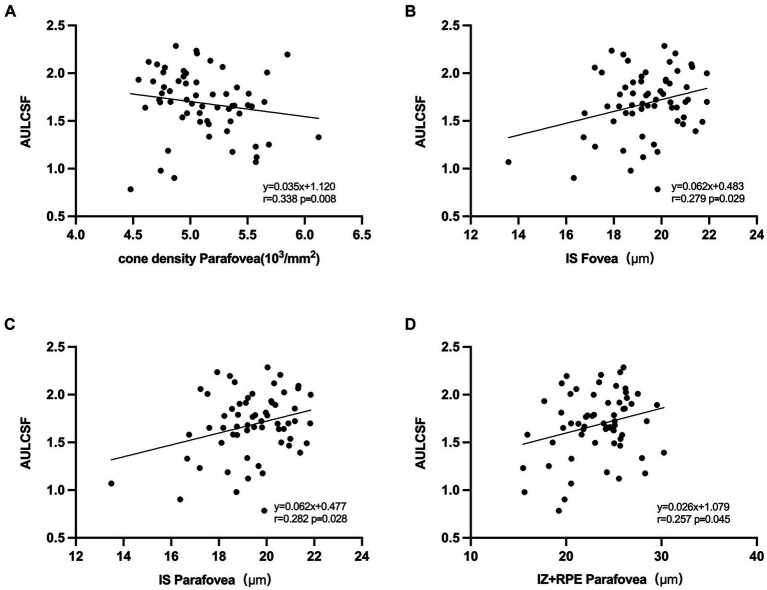
Correlation between AULCSF and retinal microstructural. **(A)** Correlation between AULCSF and cone density in the outer region. **(B)** Correlation between AULCSF and IS thickness in the inner region. **(C)** Correlation between AULCSF and IS thickness in the outer region. **(D)** Correlation between AULCSF and IZ + RPE thickness in the outer region.

In the univariate regression models (Figure 5), a larger AULCSF was significantly associated with a thinner IS and higher CVI and LA in all regions (*r* = 0.272 to 0.327, all *p* < 0.050). For the outer region, a better AULCSF was associated with a higher cone density, thicker IZ + RPE (*r* = 0.338 and 0.257, respectively, *p* < 0.050) and a shorter AL (*r* = −0.256, *p* < 0.050). The thickness of the OPL, HFL + ONL and OS were not significantly associated with CSF (all *p* > 0.050).

In the multivariate analysis, CSF was included as a dependent variable. The variables that were significantly associated with CSF in the univariate analyses were included in the multivariate model. As a result, the independent parameters OPL, HFL + ONL, IS, OS, IZ + RPE, CT, CVI, LA, and AL were dropped from the list of independent parameters. In the final model, better CSF was significantly associated with a higher cone density in the outer region (*R* = 0.338, *p* = 0.008).

### 3.6 Associations between CT and the cone distribution and retinal sublayer thickness

Thinning of the choroid was significantly correlated with cone density and regularity, the thicknesses of the IS (*r* = 0.010, all *p* < 0.050) and OPL in the inner region (*r* = 0.326, *p* < 0.050), and the thickness of the ONL in the inner region (*r* = 0.314, *p* < 0.050).

## 4 Discussion

This study used high-resolution imaging techniques such as AO and OCT to identify whether structural changes in the fundus play a role in the visual impairment characteristic of SHM eyes. All participants in this study had a best-corrected visual acuity (BCVA) of 20/20 or higher with no other complications. The results of this study revealed that patients in the SHM group had reduced IS and RPE thicknesses, reduced choroidal thickness and vascular density, but increased inner OS thickness and increased deep and superficial blood flow in the inner region of the retina, as compared to the control group. Moreover, the cone density and retinal thickness, particularly the IS and RPE layer thicknesses, were significantly correlated with reduced CSF in SHM. The results indicate that the morphologic degeneration of photoreceptors in SHM eyes was significantly correlated with the deterioration in contrast sensitivity. Since a reduction in contrast sensitivity has been widely documented in previous studies (Liu et al., 2022), early intervention to protect the photoreceptors may be critical in maintaining visual function.

It is difficult to detect visual damage in SHM because the progression of SHM and related pathological changes is slow. Visual acuity might be a poor functional endpoint as it may be unaffected until the late stages of SHM (i.e., when pathological changes or complications occur) (Iacono et al., 2017; Lee et al., 2017). Contrast sensitivity, however, captures the ability to see low and high contrast patterns, making it a more sensitive and informative index for assessing spatial vision (Arden, 1978). This is especially important when diagnosing diseases with normal visual acuity and fundus images. A reduction in CSF in young patients with SHM and normal BCVA was also found here. This result also confirms that patients with SHM have significantly reduced contrast sensitivity before visual impairment. The reason and mechanism of the decrease in the contrast sensitivity in SHM have not yet been determined, which is one of our future research directions. Moreover, both the AULCSF and Cut-off SF were significantly decreased representing a decrease in the visual function in the central area of the macula. The decline in CSF showed a strong correlation with the cone distribution, which is consistent with previous studies (Liu et al., 2022). Although the sequence of the contrast sensitivity damage and retinal microstructural changes is not yet clear, our findings provide valuable insights for further research.

The distribution of cone photoreceptors is known to be an important factor in visual dysfunction. While some previous studies have found that a lower cone distribution is associated with poorer visual function, it is unclear whether or not this is indeed the case (Woog and Legras, 2019; Cheng et al., 2021; Xin et al., 2021). According to the current study, a lower cone distribution is associated with poorer contrast sensitivity in individuals with SHM. Lower cone packing density and regularity in SHM eyes indicate potential sparseness and disordered cone arrangement within specific areas (Woog and Legras, 2019). The current study found that decreased contrast sensitivity is linked to chorioretinal degeneration, specifically, photoreceptor dysfunction. Several studies have found links between retinal function and microstructure in HM (Park et al., 2013; Wang et al., 2019; Woog and Legras, 2019; Cheng et al., 2021; Xin et al., 2021). The current study also found that increased AL was significantly associated with decreased cone density and increased cone spacing. The longer AL of myopic persons implies that the surrounding world is imaged on a larger retinal area than in non-myopic eyes. This effect might have implications for contrast sensitivity and might contribute to our understanding of the relationship between ocular optics and neurosensory function.

Moreover, it was found that in the inner and outer regions, the IS and RPE thicknesses were significantly lower in the SHM group than in the EM group. However, in the outer region, the IS thickness was significantly higher. In summary, a trend toward a significant reduction in IS and RPE thickness was found in SHM patients. This finding is also consistent with the previously reported works in the literature, in which the elongation of the eye axis in HM patients led to mechanical stretching of the retina, resulting thus in retinal thinning (Ooto et al., 2010; Yamashita et al., 2015). Retinal degeneration mainly occurs in the OS layer, a layer with many retinal glial cells. Glial cells are the supporting structures in the retina. Therefore, the changes in the retinal photoreceptor outer segment thickness observed in the current work could explain the fact that HM is a risk factor for retinal degeneration. In the present work, it was also found that the OS thickened with increasing AL. Histological findings have revealed prolonged outer segments of photoreceptors in animal models of deprivation myopia, which is consistent with the present findings (Cook et al., 1995). Nevertheless, here, no significant correlation between OS and CSF was detected. Although more in-depth studies are needed, these findings provide a novel way of thinking about retinal damage in SHM patients.

We found that in the outer region, the superficial retinal blood flow was slightly increased in the SHM group compared to the EM group, and the deep retinal blood flow was slightly decreased, but the difference was not statistically significant. Al-Sheikh et al. (2017) performed OCTA on the macula of myopic patients and found that retinal capillary density decreased to varying degrees with increasing myopia. Su et al. (2020) confirmed this idea in HM eyes. With the elongation of the eye axis, the eye is overstretched and the retinal and choroidal vessels are stretched. When the retinal and choroidal vessels are stretched, their diameters become thinner and blood flow is reduced, which will directly lead to a reduction in the vascular perfusion area. In the inner region, the superficial and deep blood flow densities were significantly increased in the SHM group compared to the EM group. We speculate that this may be due to choroidal vascular compression in patients with SHM, resulting in inadequate retinal perfusion. At the same time, the absence of blood vessels in the central macular recess promotes compensatory proliferation of the peripheral retinal capillaries into the macular region, resulting in increased density in the central macular recess region. This is consistent with the findings of Mo et al. (2017) and Milani et al. (2018), who found no significant change in the macular central recess blood flow density and reported a possible mechanism for macular central recess protection in HM. In the current study, the FAZ of the avascular macular central recess was significantly decreased in the SHM and MM/LM groups compared to the EM group, suggesting that fundus perfusion is significantly lower in HM patients than in healthy eyes (Mastropasqua et al., 2019).

Myopia microangiopathy affects the two microcirculatory systems that supply oxygen and nutrients to the neural retinal tissue in the eyes (Birol et al., 2007; Linsenmeier and Zhang, 2017). The retinal circulation provides oxygen and nutrients to the inner retinal tissue while the choroidal circulation, which includes the photoreceptor layer via the choriocapillaris, provides oxygen and nutrients to the outer retinal layer (Wangsa-Wirawan and Linsenmeier, 2003). The significant choroid thickening in myopia and its relationship with visual function has been extensively studied in recent years (Gupta et al., 2015; Zaben et al., 2015). The choroid provides most of the oxygen demand of photoreceptors (Linsenmeier and Braun, 1992; Wangsa-Wirawan and Linsenmeier, 2003; Birol et al., 2007). Gupta et al. (2017) reported reductions in choroidal LA and SA along with thinning of the ChT in HM eyes, which is consistent with the current findings. We found that reductions in choroidal vasculature and choriocapillaris blood perfusion were positively correlated with AL. These results suggest the presence of decreased choroidal circulation in SHM. The thinning of the choroid might result in a lack of oxygen, which is necessary for photoreceptor metabolism and photosensitivity. This could explain the significant correlations between thinning of the choroid and the cone distribution, IS, OS, and IZ + RPE layer thicknesses, and visual dysfunction. As a result, improving the photoreceptor microenvironment and preventing degeneration could be a new target for the treatment of visual dysfunction in SHM.

The current study has several limitations that should be noted. First, this study mainly recruited individuals aged 18–30 years, which is not representative of the general population. Second, because this was a cross-sectional study, the causal relationship between photoreceptor morphology and contrast sensitivity cannot be determined. Additional longitudinal studies are needed to investigate the progression of myopia and these causal relationships. Third, this study only examined a small area of the macula. Future studies should include more extensive field imaging and analysis techniques to confirm the photoreceptor alterations and dysfunction.

In conclusion, changes in the photoreceptor distribution, retinal and choroid parameters, and CSF were observed with increased degrees of myopia. This study discovered a link between the retinal microstructural and visual function in a myopic sample. A reduced cone density was positively correlated with a reduced CSF in SHM, indicating that changes in cone photoreceptors can decrease contrast sensitivity. Longitudinal research is needed to determine the possible causal nature of these associations. Early monitoring of contrast sensitivity and attention to changes in retinal microstructure may be crucial to preventing the development of pathological myopia in people with SHM.

## Data availability statement

The raw data supporting the conclusions of this article will be made available by the authors, without undue reservation.

## Ethics statement

The studies involving humans were approved by Chinese Clinical Trials Registry (Registration No. ChiCTR2000040926). The studies were conducted in accordance with the local legislation and institutional requirements. The participants provided their written informed consent to participate in this study. Written informed consent was obtained from the individual(s) for the publication of any potentially identifiable images or data included in this article.

## Author contributions

JW: Investigation, Methodology, Writing – review & editing, Conceptualization, Data curation, Formal analysis, Project administration, Resources, Software, Writing – original draft. XL: Methodology, Writing – original draft, Investigation, Project administration, Writing – review & editing, Funding acquisition, Supervision. JH: Methodology, Writing – review & editing, Data curation, Formal analysis, Software. RD: Data curation, Formal analysis, Methodology, Writing – review & editing, Project administration. SZ: Data curation, Formal analysis, Methodology, Writing – review & editing. YC: Data curation, Methodology, Writing – review & editing. ZC: Data curation, Methodology, Writing – review & editing. YW: Data curation, Methodology, Writing – review & editing. YR: Data curation, Methodology, Writing – review & editing. QL: Data curation, Methodology, Writing – review & editing. JQ: Methodology, Funding acquisition, Investigation. XM: Methodology, Writing – review & editing, Funding acquisition, Investigation.
